# Broad-spectrum Delta-BA.2 tandem-fused heterodimer mRNA vaccine delivered by lipopolyplex

**DOI:** 10.1371/journal.ppat.1012116

**Published:** 2024-04-01

**Authors:** Pei Du, Lei Huang, Yi Fang, Fanfan Zhao, Qianyun Li, Xuehui Ma, Ruiqi Li, Qian Chen, Haifa Shen, Qihui Wang, Hangwen Li, George Fu Gao

**Affiliations:** 1 CAS Key Laboratory of Pathogen Microbiology and Immunology, Institute of Microbiology, Chinese Academy of Sciences (CAS), Beijing, China; 2 Stemirna Therapeutics, Shanghai, China; 3 Medical School, University of Chinese Academy of Sciences, Beijing, China; 4 Faculty of Health Sciences, University of Macau, Macau, China; National Institute of Allergy and Infectious Diseases, UNITED STATES

## Abstract

Since the beginning of the coronavirus disease 2019 (COVID-19) pandemic, severe acute respiratory syndrome coronavirus 2 (SARS-CoV-2), the causative agent of COVID-19, continues to mutate and generates new variants with increasingly severe immune escape, urging the upgrade of COVID-19 vaccines. Here, based on a similar dimeric RBD design as our previous ZF2001 vaccine, we developed a novel broad-spectrum COVID-19 mRNA vaccine, SWIM516, with chimeric Delta-BA.2 RBD dimer delivered by lipopolyplex (LPP). Unlike the popular lipid nanoparticle (LNP), this LPP-delivered mRNA expresses only in the injection site, which avoids potential toxicity to the liver. We demonstrated the broad-spectrum humoral and cellular immunogenicity of this vaccine to Delta and Omicron sub-variants in naïve mice and as booster shots. When challenged with Delta or Omicron live virus, vaccinated human angiotensin-converting enzyme (hACE2) transgenic mice and rhesus macaques were both protected, displaying significantly reduced viral loads and markedly relieved pathological damages. We believe the SWIM516 vaccine qualifies as a candidate for the next-generation broad-spectrum COVID-19 vaccine.

## Author summary

mRNA vaccines have demonstrated their advantages in immunogenicity and development speed during the coronavirus disease 2019 (COVID-19) pandemic. However, the significant immune escape caused by the evolution of the severe acute respiratory syndrome coronavirus 2 (SARS-CoV-2) has presented a considerable challenge to the effectiveness of COVID-19 vaccines. Moreover, concerns have arisen about the potential liver toxicity associated with the lipid nanoparticle (LNP) used in approved mRNA vaccines, raising questions about their safety. To address these challenges, we have developed the SWIM516 vaccine, which utilizes a chimeric Delta-BA.2 RBD dimer immunogen to provide broad-spectrum protection and a lipopolyplex (LPP) delivery system to enhance safety. This vaccine has demonstrated its ability to induce broad-spectrum immunogenicity in mice and offers protection to both mice and rhesus macaques when exposed to live SARS-CoV-2 challenges, qualifying it as a next-generation broad-spectrum COVID-19 vaccine.

## Introduction

The coronavirus disease 2019 (COVID-19) pandemic has led to over 7 million deaths and countless economic losses worldwide (https://www.who.int/). Building herd immunity through vaccination is the most efficient method for preventing the spread of viruses [1–3]. However, over three years since the beginning of the pandemic, the severe acute respiratory syndrome coronavirus 2 (SARS-CoV-2) continues to mutate, bringing new variants with increasing capabilities of immune escape, such as the variants of concern (VOCs) and variants of interest (VOIs) defined by the World Health Organization [4–7]. As a result, COVID-19 vaccines based on immunogens from the SARS-CoV-2 prototype (PT) have gradually become ineffective against the prevalent Omicron sub-variants [8–10], which requires the development of a new generation of broad-spectrum vaccines to protect against the current and future SARS-CoV-2 variants. To date, several vaccines targeting Omicron sub-variants have been approved, including bivalent mRNA vaccines and XBB.1.5 monovalent vaccines by Moderna and Pfizer/BioNTech, an mRNA vaccine SY6006 by CSPC Pharmaceutical Group and two multivalent protein subunit vaccines SCTV01E (Shenzhou Cell) and Coviccine (WestVac Biopharma). These vaccines, containing immunogens from BA.1, BA.5 or XBB.1.5, have displayed potent efficacy against Omicron sub-variants as boosters [11–15].

Previously, our group developed a universal tandem dimeric RBD design of a vaccine against betacoronavirus, which led to the world’s first COVID-19 protein subunit vaccine, ZF2001, that has been approved in China, Uzbekistan, Indonesia, and Columbia [16–18]. Recently, we demonstrated that as with the immunogen of protein subunit and mRNA vaccines, a chimeric RBD dimer design with a Delta RBD and an Omicron (BA.1) RBD could elicit broad-spectrum immunogenicity against multiple SARS-CoV-2 variants in naïve mice and mice prime-vaccinated with inactivated vaccines [19–21]. However, with the shift of circulating Omicron sub-variants, the RBDs of immunogens should be updated accordingly.

The delivery system is crucial for mRNA vaccines, as it casts a great impact on the location and efficiency of *in vivo* mRNA expression [22–25]. To date, the most popular delivery system for mRNA vaccines is lipid nanoparticle (LNP) which has been used in Moderna’s mRNA-1273 and BioNTech’s BNT162b2 [26,27]. However, the currently approved LNP formula tends to deliver mRNA into the liver, which poses the potential risk of liver toxicity to vaccinees [24]. Alternative to LNP, a core-shell structured lipopolyplex (LPP) is also an effective way to deliver mRNA vaccine [28,29]. Compared with LNP, LPP mostly delivers mRNA to be expressed at the injection site after intermuscular inoculation instead of liver cells and displays adjuvant activity by activating the Toll-like receptor signaling pathway in dendritic cells, which improves the safety and immunogenicity of LPP-delivered vaccine simultaneously [28].

**Fig 1:**
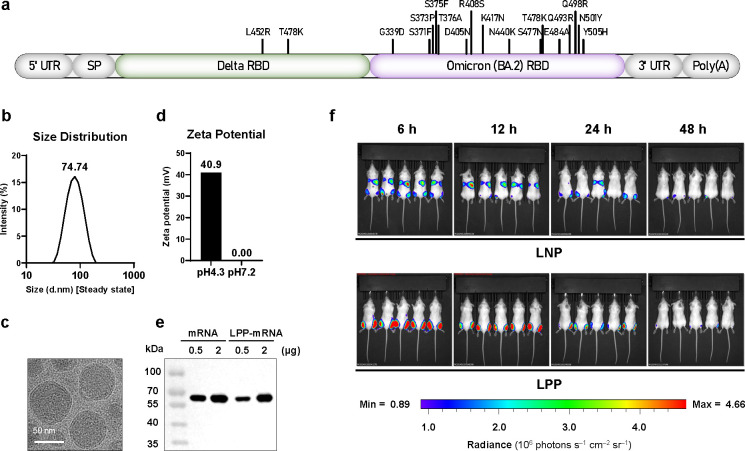
Design and *in vitro* characterization of the SWIM516 mRNA vaccine. (**a**) Schematic of the design of the Delta-BA.2 mRNA vaccine (SWIM516). The amino acid substitutions above the column indicate the mutations in Delta and BA.2 RBDs compared with PT RBD. (**b**) Particle size distributions of LPP characterized by dynamic light scattering. The number indicates z-average. (**c**) Cryo-electron microscopy image of lipopolyplex (LPP). Scale bar, 50 nm. (**d**) Zeta potential for LPP at pH 4.3 and 7.2 in phosphate saline buffer (PBS). (**e**) Naked or LPP-encapsulated mRNA was transfected into HEK293T cells. The expression of Delta-BA.2 in the supernatant was analyzed by Western blot. (**f**) Locations of LNP- and LPP-delivered luciferase expression in *vivo*. mRNAs encoding luciferase were encapsulated by LNP or LPP before injection in both legs of BALB/c mice. The bioluminescence images of mice were captured at the indicated time points post-injection.

In the present study, we provide a novel mRNA vaccine (named SWIM516) with a Delta-BA.2 chimeric RBD dimer delivered by LPP (Fig 1A). We evaluated the broad-spectrum immunogenicity of the SWIM516 vaccine to SARS-CoV-2 PT, Delta and Omicron sub-variants in naïve mice and mice prime-vaccinated with inactivated or protein subunit vaccine. We also demonstrated the protection against live viruses in mice (Delta and BA.1) and rhesus macaques (BA.1) by SWIM516. This vaccine demonstrated a great potential to protect against a wide range of Omicron sub-variants, including BA. 5 and BF.7, which qualified it as a candidate for next-generation broad-spectrum COVID-19 vaccines.

## Results

### Design and *in vitro* characterization of the SWIM516 mRNA vaccine

To design the SWIM516 mRNA vaccine, the SARS-CoV-2 Delta and BA.2 RBDs were chosen and dimerized as a Delta-BA.2 tandem repeat (Fig 1A). To prepare the SWIM516 vaccine, the mRNA transcripts containing the 5′-untranslated region, the signal peptide, the Delta-BA.2 RBD-dimer coding sequence and 3′-untranslated region were transcribed by *in vitro* transcription, modified by 5′ capping and encapsulated by LPP. Then, the quality control of LPP encapsulation was conducted by examining the size distribution and zeta potential with dynamic light scattering. We discovered that LPP displayed a similar average diameter (~75 nm), polydispersity index (0.1115), and round shape similar to LNP (Fig 1B and 1C). Moreover, the surface charge of LPP was positive at pH 4.3 and become neutral at pH 7.2, demonstrating that LPP could enter cells with a mechanism similar to that of LNP (Fig 1D).

Next, we characterized the *in vitro* and *in vivo* expression of SWIM516, and visualized it using cryo-electron microscopy. The *in vitro* expression of SWIM516 was characterized by transfection of naked or LPP-encapsulated mRNA transcripts into HEK293T cells and examination of the translated immunogen in the supernatant. Western blotting showed that both naked mRNA and the LPP-encapsulated mRNA vaccine could express the Delta-BA.2 RBD dimer as a single band at its theoretical size in a dose-dependent manner, indicating the integrity of the immunogen translated *in vivo* (Fig 1E). The expression location of the LPP-delivered SWIM516 vaccine in mice was visualized by *in vivo* imaging of the bioluminescence of mRNA-expressed luciferase. Our results indicated that, unlike LNP, LPP-delivered mRNA expression was concentrated in the muscle instead of partially expressing in the liver, and the expression can last approximately 48 hours (Fig 1F).

### Evaluation of the immunogenicity of the SWIM516 vaccine

**Fig 2:**
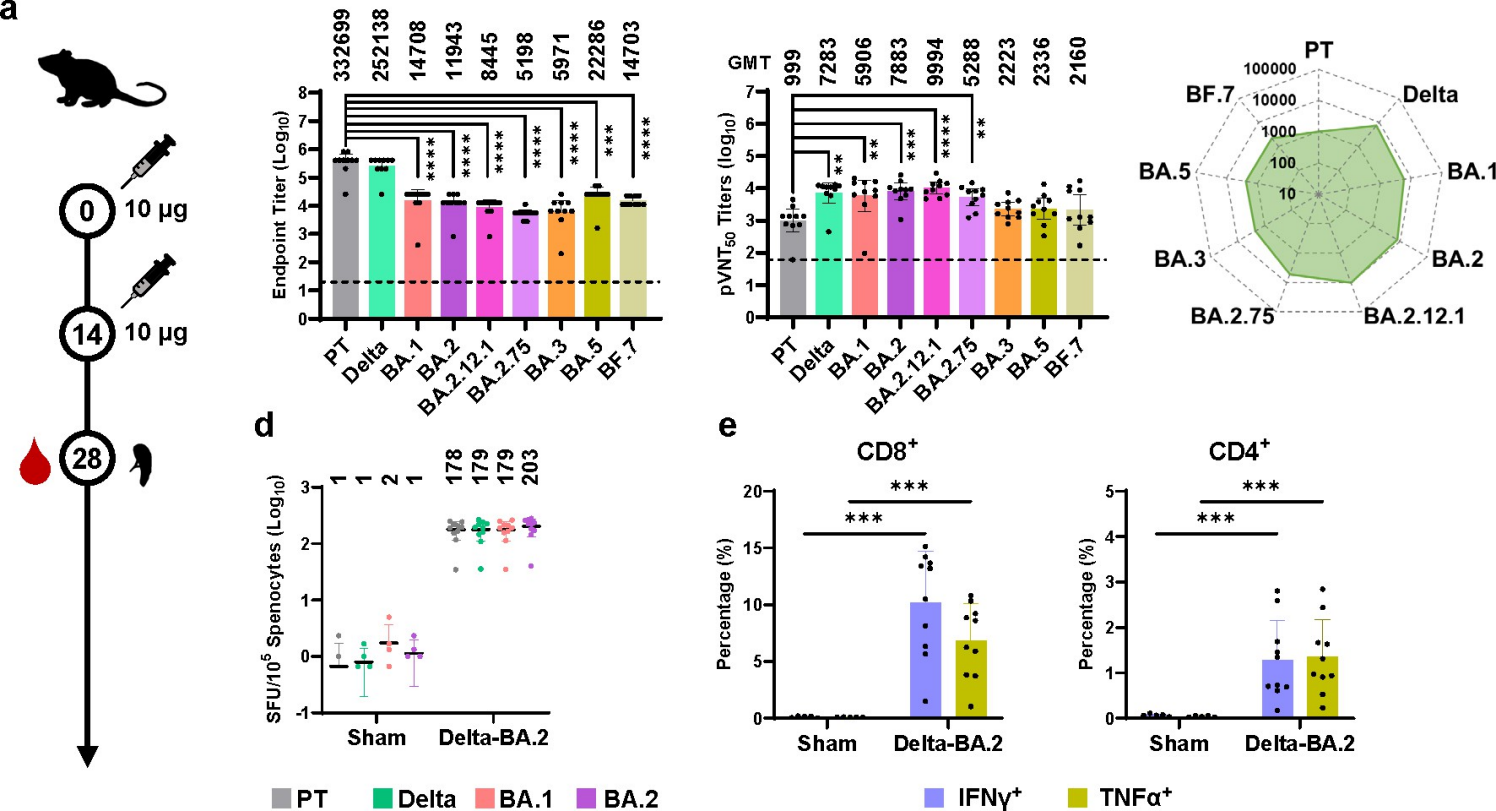
Evaluation of the immunogenicity of the SWIM516 vaccine. (**a**) Mice immunization and sample collection schedule for evaluating the immunogenicity of SWIM516 vaccine. (**b**) Titers of IgG specific to the RBDs of the indicated SARS-CoV-2 variants on day 28. Numbers on top indicate the average IgG titers of each group. Dashed line indicates starting dilution (20 folds). Data are shown as geometric mean titer (GMT) ± 95% confidence interval (CI). (**c**) NT_50_ of neutralizing antibodies against the pseudotyped viruses (pVirus) of indicated SARS-CoV-2 variants. Numbers on top indicate the GMT of each group. Radar chart was drawn based on GMT. Dashed line indicates starting dilution (60 folds). Data are shown as GMT ± 95% CI. (**d**) ELISpot assay quantifying the IFNγ-secreting splenocytes after re-stimulation by RBD peptide pool of indicated SARS-CoV-2 variants. Data are shown as means ± SD (standard deviation). Numbers on top indicate average SFU/10^5^ splenocytes. (**e**) ICS assays quantifying the proportions of IFNγ- and TNFα-secreting CD8^+^ and CD4^+^ T cells stimulated by BA.2 RBD peptide pool. Data are shown as means ± SD. ELISA and pseudovirus neutralization assays were repeated twice. All statistical significances were calculated by Mann-Whitney test (*, p<0.05; **, p<0.01; ***, p<0.001, ****, p<0.0001).

To examine the immunogenicity of the SWIM516 vaccine, we immunized groups of C57BL/6 mice (N = 10) twice (10 μg/dose) at a 14-day interval and collected serum samples as well as splenocytes 14 days after the second dose (day 28) (Fig 2A). Enzyme-linked immunosorbent assay (ELISA) data showed that the serum samples contained high titers of antibodies specific to the RBDs of different SARS-CoV-2 variants, including PT, Delta, BA.1, BA.2, BA.2.12.1, BA.2.75, BA.3, BA.4/5 and BF.7 (Fig 2B). Moreover, neutralization assays revealed that the neutralizing antibodies (NAbs) elicited by the SWIM516 vaccine could effectively neutralize pseudotyped viruses of PT (GMT:999), Delta (GMT:7283), BA.1 (GMT:5906), BA.2 (GMT:7883), BA.2.12.1 (GMT:9994), BA.2.75 (GMT:5288), BA.3 (GMT:2223), BA.4/5 (GMT:2336) and BF.7 (GMT:2160) variants (Fig 2C). As demonstrated by the radar chart, the SWIM516 vaccine could elicit broad-spectrum humoral immunogenicity against multiple SARS-CoV-2 variants, including the Omicron sub-variants BA.5 and BF.7 [30]. Notably, although these serum samples contained significantly higher titers of antibodies specific to the RBDs of the PT compared to those targeting the Omicron sub-variants, they demonstrated superior efficacy in neutralizing the pseudotyped viruses of Omicron sub-variants, especially BA.2 and its derivatives. (Fig 2B and 2C).

In addition to humoral immunogenicity, the cellular immunogenicity of the SWIM516 vaccine was also evaluated with splenocytes. After re-stimulation with peptide pools of different RBDs (PT, Delta, BA.1, or BA.2), enzyme-linked immune absorbent spot (ELISpot) assay showed that, with SWIM516 vaccination, the number of IFN-γ^+^ spots in all four groups dramatically increased to a higher level (Fig 2D). Interestingly, the T cell activation levels after stimulation by different peptide pools were almost identical. Using *in silico* analysis, we predicted multiple conserved peptides in the RBDs of PT, Delta, BA.1 and BA.2 that could have high binding affinity with MHC class I (S2 Fig), indicating SWIM516 could elicit a broad-spectrum T cell response to multiple SARS-CoV-2 variants. The intracellular cytokine staining (ICS) assay also consistently demonstrated significantly increased percentages of IFN-γ^+^ and TNF-α^+^ populations in both CD8^+^ and CD4^+^ cells after re-stimulation with the BA.2 RBD peptide pool (Fig 2E). These data verified the cellular immunogenicity of the SWIM516 vaccine in response to both BA.1 and BA.2.

Notably, LPP-delivered mRNA vaccine exhibited comparable humoral and cellular immunogenicity as LNP-delivered mRNA vaccines. By delivering a mRNA vaccine using LNP or LPP in a side-by-side comparison experiment, neutralization and ELISpot assays demonstrated similar levels of NAbs and similar numbers of IFN-γ^+^ spots, respectively (S1 Fig).

### Evaluation of the immunogenicity of SWIM516 as boosters

**Fig 3:**
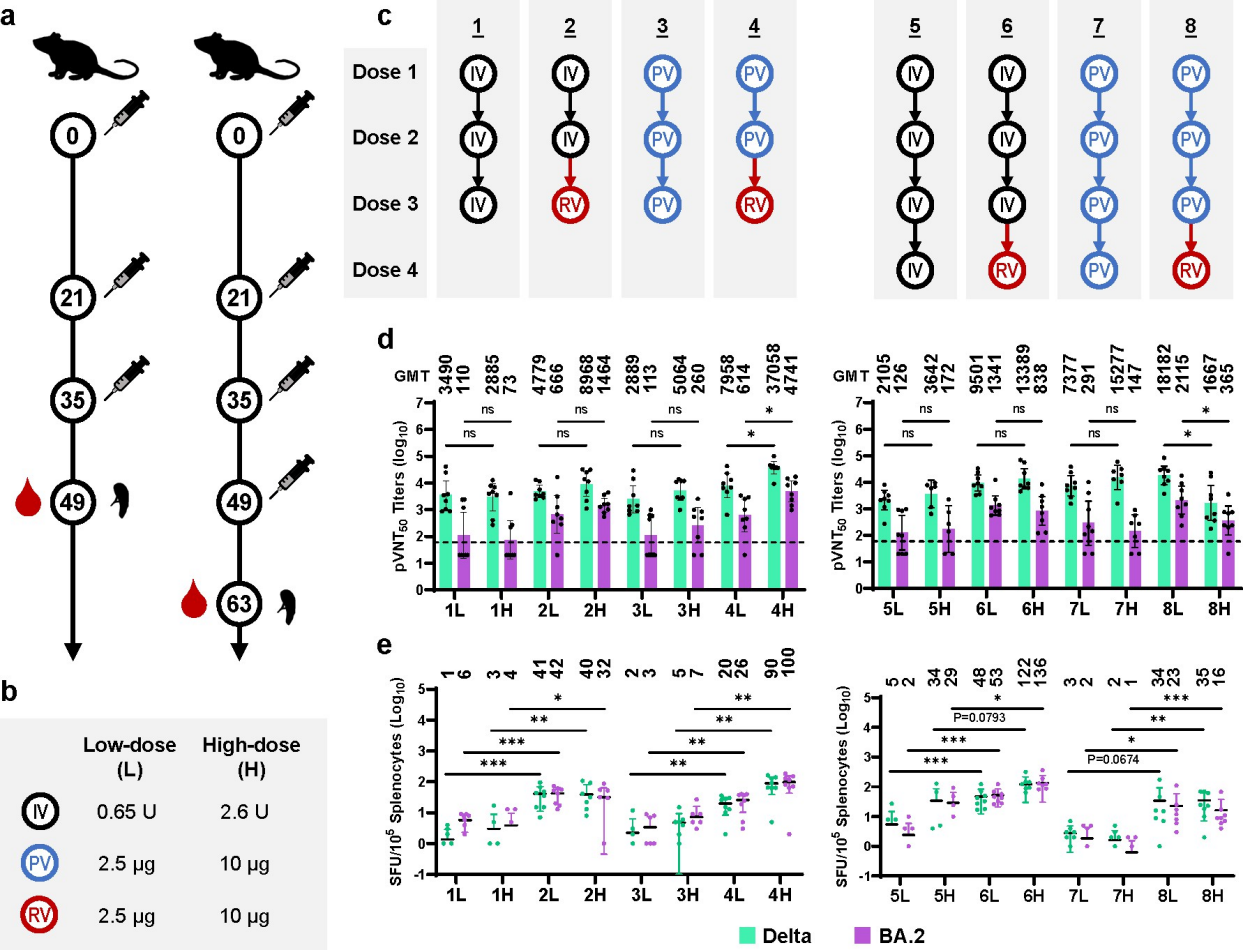
Evaluation of the immunogenicity of SWIM516 as boosters. (**a**) Mice immunization and sample collection schedule for evaluating the immunogenicity of SWIM516 vaccine as the third or fourth shot. (**b**) The low and high dosages of inactivated vaccine (IV), protein subunit vaccine (PV) and mRNA vaccine (RV) used in the prime-boost immunization protocols. (**c**) The prime-boost immunization protocols of three-dose (left) or four-dose (right) groups. Underlined numbers on top indicate group numbers. The numbers in groups names of (d) and (e) correspond with the group numbers in (c), letter L and H indicate low-dose and high-dose groups, respectively. (**d**) NT_50_ of neutralizing antibody against Delta or BA.2 pseudotyped viruses in three-dose (left) or four-dose (right) groups. Numbers on top indicate GMT. Dashed line indicates starting dilution (60 folds). Data are shown as GMT ± 95% CI. Pseudovirus neutralization assays were repeated twice. (**e**) ELISpot assay quantifying the IFNγ-secreting splenocytes after re-stimulation by Delta or BA.2 RBD peptide viruses in three-dose (left) or four-dose (right) groups. Data are shown as means ± SD. Numbers on top indicate average SFU/10^5^ splenocytes. All statistical significances were calculated by Mann-Whitney test (*, p<0.05; **, p<0.01; ***, p<0.001).

Based on the evaluation in naïve mice, we further designed a prime-boost experiment to assess the efficacy of SWM516 vaccines as booster shots following the inactivated vaccines (IV) or protein subunit vaccines (PV), which reflects the vaccination status in China and many other countries (Fig 3A). Groups of C57BL/6 mice (N = 8) were prime-vaccinated with two or three doses of IV (0.65 U or 2.6 U/dose) or PV (2.5 μg or 10 μg/dose) at a 14-day interval, and boosted with the SWIM516 mRNA vaccine (RV, 2.5 μg or 10 μg/dose) 14 days later (Fig 3B and 3C). IV or PV were also vaccinated in parallel as controls (Fig 3C). Serum samples and splenocytes were collected 14 days post the last dose (day 49 or day 63) and subjected to neutralization assays and ELISpot assays, respectively.

Neutralization assay results showed that the levels of NAbs against Delta or BA.2 pseudotyped viruses in groups vaccinated with SWIM516 as the booster shot were higher than that in groups with IV or PV boosters (Fig 3D). Compared with three doses of IV (2.6 U/dose, GMT:73), a third dose of SWIM516 (10 μg, GMT:1464), following two doses of IV, elicited 20-folds higher BA.2-specific NAbs. Similarly, a third dose of SWIM516 (10 μg, GMT:260), following two doses of PV, lead to an 18.2-fold increase of BA.2-specific NAbs compared with three doses of PV (10 μg/dose, GMT:4741) (Fig 3D). Notably, the SWIM516-elicited NAbs demonstrated a clear dose-dependent trend in the three-dose groups, as the high-dose groups (10 μg SWIM516) displayed significantly higher Delta- or BA.2-specific NAbs than the low-dose groups (2.5 μg SWIM516) (Fig 3D). Interestingly, a fourth dose of low-dose SWIM516 (2.5 μg) elicited higher levels of NAbs than a fourth dose of IV or PV, but a high-dose SWIM516 (10 μg) as the fourth dose resulted in significantly reduced NAbs levels against both Delta and BA.2 compared with the low-dose group (Fig 3D). This result is consistent with our prior observation that exceedingly high-dose vaccination can lead to reduced level of NAbs [16], possibly due to immune tolerance generated by repeated vaccination [31]. The mechanism of such a phenomenon deserves further study. Additionally, the cellular immunogenicity of SWIM516 as booster shots was also evaluated by ELISpot assays. Similar to NAbs levels, the numbers of IFN-γ^+^ spots after re-stimulation of Delta or BA.2 RBD peptide pools were also significantly increased by switching the third or fourth dose from IV or PV to SWIM516 (Fig 3E). These data demonstrated that SWIM516 could be a better choice as booster shots than the PT-based inactivated vaccine or protein subunit vaccine.

### Evaluation of the protective efficacy of SWIM516 in mice and rhesus macaques

**Fig 4:**
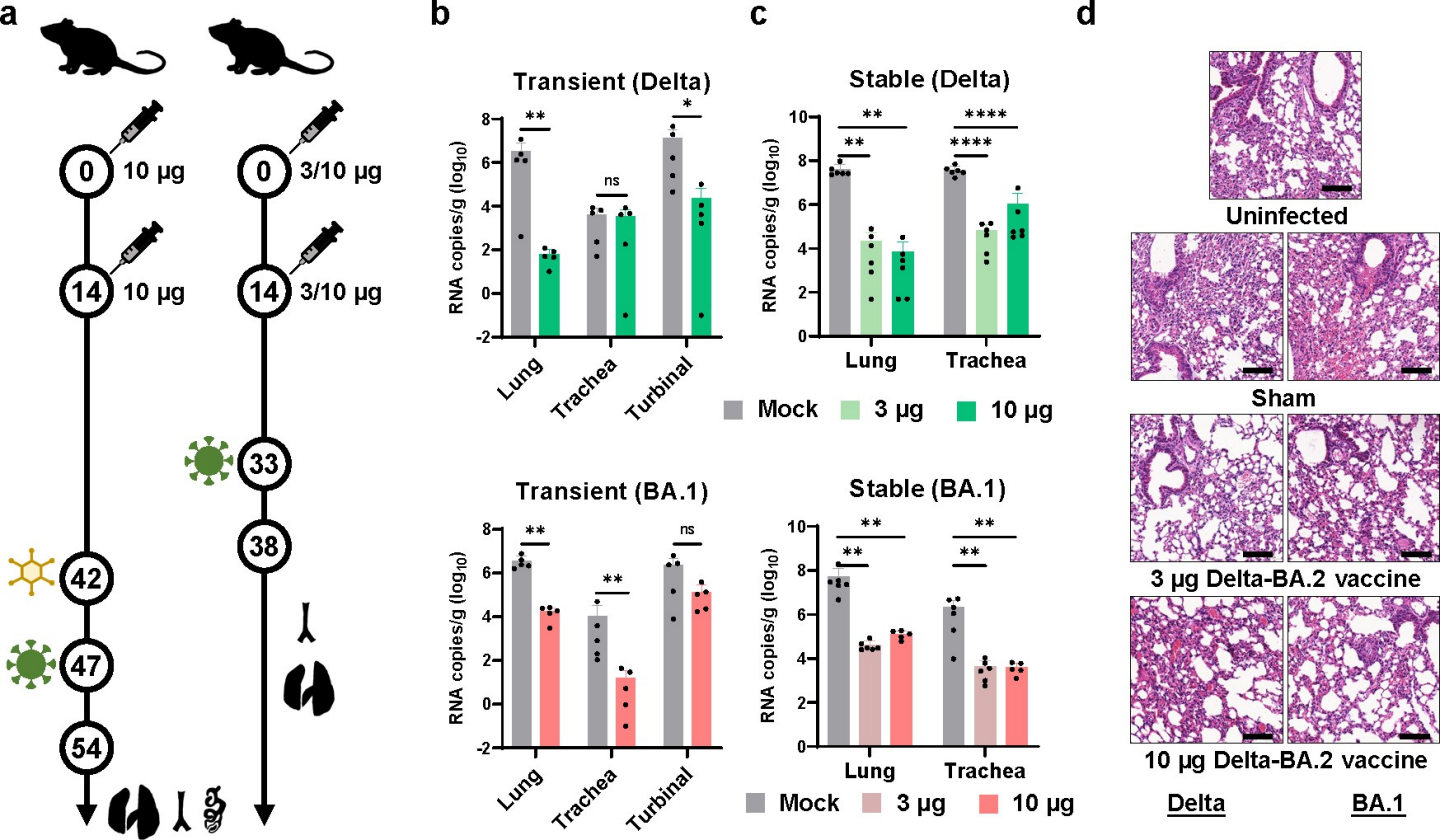
Evaluation of the protective efficacy of SWIM516 in mice. (**a**) Mice immunization, challenge and sample collection schedule for evaluating the protective efficacy of the SWIM516 vaccine. (**b**) Titration of viral loads in lung, trachea or turbinal after Delta (top) or BA.1 (bottom) challenge of transient Ad5-hACE2 mice. (**c**) Titration of viral loads in lung and trachea after Delta (top) or BA.1 (bottom) challenge of hACE2 transgenic mice. Data in (b) and (c) are shown as means ± SD. (**d**) Pathological analysis of lung tissues of mice after Delta or BA.1 challenge of hACE2 transgenic mice. Uninfected indicate mice without viral challenge. Black bars represent 100 μm. All statistical significances were calculated by Student’s T test (*, p<0.05; **, p<0.01; ***, p<0.001; ****, p<0.0001).

To further evaluate the protective efficacy of the SWIM516 vaccine, we challenged immunized mice with the SARS-CoV-2 virus using previously established mouse models transiently or stably expressing human angiotensin-converting enzyme (hACE2) [32,33]. For the transient hACE2 mice model, we immunized C57BL/6 mice twice (10 μg/dose) on days 0 and 14, transduced them with recombinant type 5 adenovirus expressing hACE2 and challenged them with 1×10^5^ TCID_50_ of BA.1 or Delta variant via the intranasal route. Lung, trachea, and turbinal tissues were harvested three days post-challenge (Fig 4A). High levels of both Delta and BA.1 virus were detected in all tissues of unvaccinated mice. In comparison, the vaccinated group showed four and two orders of magnitude lower levels of Delta and BA.1 in lung tissues, respectively (Fig 4B). Notably, in tracheal tissues, vaccination also reduced the BA.1 viral load by approximately 1000-fold, which is a significant finding as Omicron was reported to primarily replicate in the respiratory tract [34].

For hACE2 transgenic mice, two different doses of vaccine were injected to investigate whether the protection against BA.1 or Delta was dose dependent. Mice immunized twice with 3 μg or 10 μg per dose were challenged intranasally with 1×10^5^ TCID_50_ of BA.1 or Delta variant. Lung and tracheal tissues were collected five days post-challenge (Fig 4C). Titration of viral loads showed that, after challenging by either Delta or BA.1, vaccination led to a two to four orders of magnitude decrease in viral load in both lung and tracheal tissues, but a higher dose of vaccination did not lead to significantly decreased viral load compared to the low dose group, indicating that low dose could provide saturated protection (Fig 4C). In addition, the pathological analysis also displayed markedly relieved pathological damage and inflammation caused by SARS-CoV-2 infection in vaccinated mice, despite existing signs of cellular infiltration (Fig 4D and S1 Table). Together, these results verified the protection against both Delta and Omicron variants by the SWIM516 vaccine.

**Fig 5:**
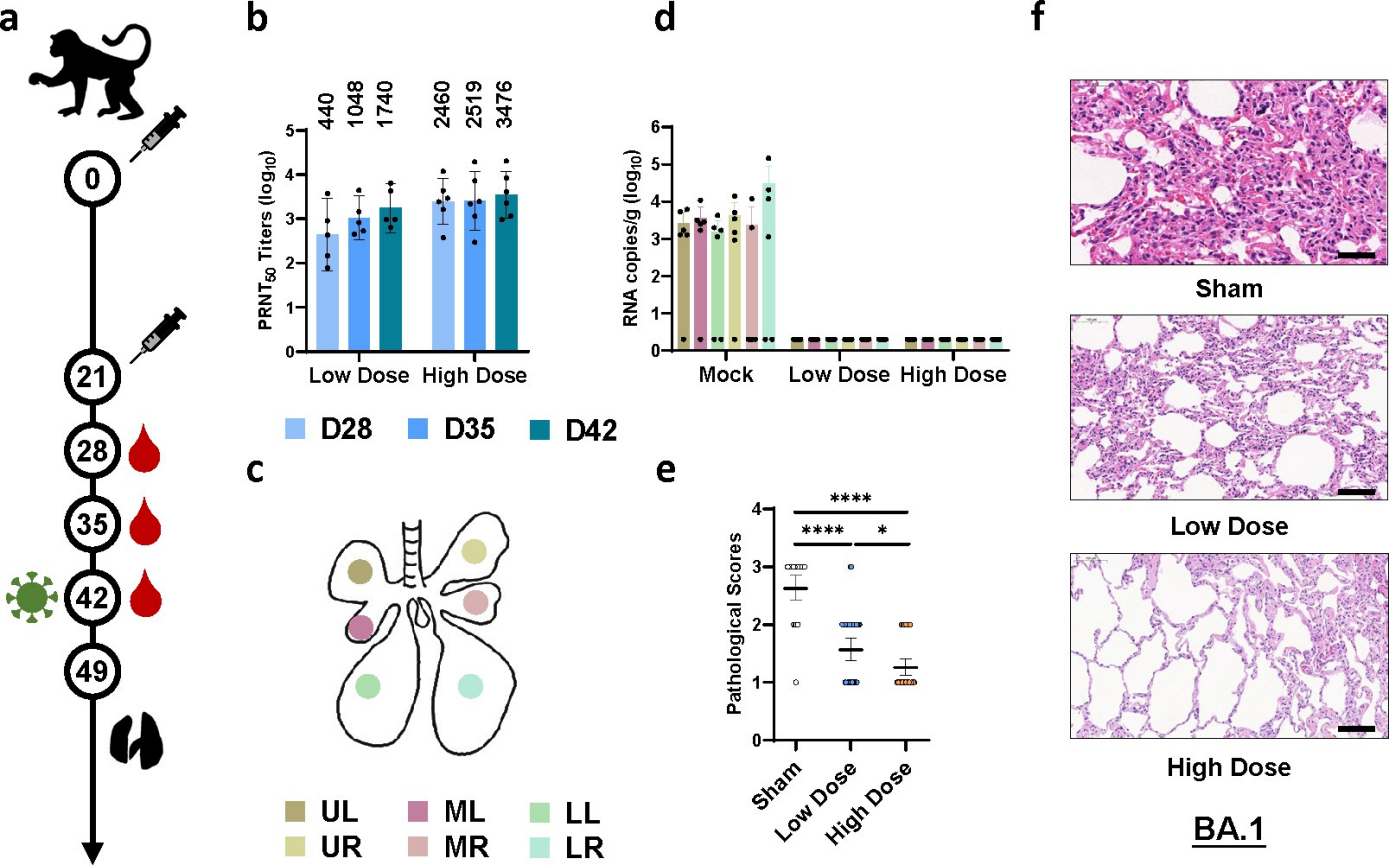
Evaluation of the protective efficacy of SWIM516 in rhesus macaques. (**a**) Rhesus macaques immunization, challenge and sample collection schedule for evaluating the protective efficacy of SWIM516 vaccine. (**b**) PRNT_50_ of neutralizing antibody against BA.1. Numbers on top indicate GMT, data are shown as GMT ± 95% CI. (**c**) Diagram of samples collected in different lobes of rhesus macaque lungs after BA.1 challenge. Legend indicated the lobe of lungs (UL: upper left; ML: middle left; LL: lower left; UR: upper right; MR: middle right; LR: lower right). (**d**) Quantitation of viral load in rhesus macaque lung samples, data are shown as mean ± SD. (**e**) Pooled analyses of pathological scores for lung tissues of macaques after BA.1 challenge, data are shown as GMT ± 95% CI. Statistical significances were calculated by Mann-Whitney test (*, p<0.05; ****, p<0.00001). (**f**) Pathological analysis of lung tissues of rhesus macaque after BA.1 challenge. Black bars represent 100 μm.

Finally, we evaluated the protective efficacy of the SWIM516 vaccine in non-human primates. Rhesus macaques were immunized twice on day 0 and day 21 with low dose (25 μg/dose), high dose (45 μg/dose) vaccine or placebo, and bled on day 28, day 35 and day 42. Then, each monkey was challenged with 1×10^5^ TCID_50_ of BA.1 on day 42 and euthanized after 7 days for lung histopathology (Fig 5A). NAbs were detected in the sera of both low-dose and high-dose groups, and a higher dose of vaccination did lead to higher levels of NAbs (Fig 5B). Moreover, in samples collected in each lung lobe of rhesus macaques (Fig 5C), vaccination of either low dose or high dose SWIM516 lead to complete clearance of virus (Fig 5D), and the pathological damage and inflammation were also significantly relieved (Fig 5E, 5F and S2–S4 Tables).

## Discussion

In the past years, the continuously emerging Omicron sub-variants displayed escalated capability of immune escape, which has gradually rendered the widely administered PT-based vaccines ineffective, urging the development of a new generation of broad-spectrum COVID-19 vaccines to protect against a broad range of SARS-CoV-2 variants for the control of COVID-19 pandemic worldwide [4–6,8–10,35,36]. Moreover, as the PT-based inactivated vaccine (*e*.*g*. BBIBP-CorV) and protein subunit vaccine (*e*.*g*. ZF2001) have been administered in many countries, it is also important to examine the immunogenicity of new COVID-19 vaccines as booster shots following these earlier vaccines, because pre-existing immunity is known to affect vaccinees’ immune response to booster vaccines [21,37].

Currently, the widely administered mRNA vaccines utilize LNP as the delivery system. However, LNP-encapsulated mRNA vaccines have been reported to express in liver cells, which brings potential side effect caused by liver-toxicity and limits the application of the mRNA vaccines [24]. The reason for this effect can partially be attributed to the interaction of LNP with apolipoprotein E (apoE) that enhances LNP entry into primary hepatocytes through the low-density lipoprotein (LDL) receptor (LDLR) [38]. To address this problem, the LPP-delivery system provides a solution. With a core-shell structure markedly different from that of LNP, LPP manages to deliver mRNAs to be expressed mostly in the muscle of the injection site with only a small proportion expressing in spleen and lymph node, as demonstrated by *in vivo* and *ex vivo* fluorescent images [29,39] (Fig 1F). Consequently, the LPP-encapsulated vaccines may have the advantage to the safety of mRNA vaccine by avoiding liver toxicity. So far, the safety of LPP has been verified by SW-BIC-213, an LPP-delivered mRNA vaccine evaluated in clinical trials and granted EUA in Laos [40–42]. Moreover, LPP-delivered mRNA vaccine could elicit similar humoral and cellular immunogenicities as LNP-delivered ones in side-by-side comparisons, suggesting LPP can be an alternative to LNP (S1 Fig) [24].

Here, we provide a new LPP-delivered COVID-19 mRNA vaccine, SWIM516, with chimeric Delta-BA.2 RBD dimer as immunogen based on our dimeric RBD design [16,19–21]. The mRNA expression of this vaccine is limited in the injection site, highlighting its potential advantage in safety. The SWIM516 vaccine expands our lineage of tandem repeat RBD-dimer designs that includes the protein subunit vaccine ZF2001 [16–18], the chimeric Delta-BA.1 protein subunit vaccine [19] and Delta-BA.1 RBD-dimer mRNA vaccine [20,21]. As expected, the SWIM516 vaccine demonstrated broad-spectrum immunogenicity to the SARS-CoV-2 PT, Delta VOCs and a wide range of Omicron sub-variants in mice including BF.7 [30], as well as high protective efficacy in mice and rhesus macaques against the challenge of live SARS-CoV-2 virus. SWIM516 is also effective as a booster shot following PT-based inactivated or protein subunit COVID-19 vaccines, making it suitable for the population in China and many other countries.

Although we were unable to conduct a viral challenge with the BA.2 live virus due to limited resources, we found that the levels of neutralizing antibodies against BA.2 and BA.1 elicited by the SWIM516 vaccine were comparable, suggesting that SWIM516 could provide similar levels of protection against BA.2 and BA.1. In addition, despite markedly relieved pathological damage signs of cellular infiltration can still be observed in the lungs of mice and rhesus macaques vaccinated with SWIM516.

In summary, we developed a new COVID-19 mRNA vaccine with the RBDs of Delta and BA.2 as an immunogen and demonstrated its broad-spectrum immunogenicity to SARS-CoV-2 PT, Delta, and multiple Omicron sub-variants, as well as its protection against live virus in mice (Delta and BA.1) and rhesus macaques (BA.1). This vaccine demonstrated a great potential to protect against a wide range of Omicron sub-variants, including BA.2, and BA.5, BF.7. We believe that it qualifies as a candidate for next-generation broad-spectrum COVID-19 vaccines.

## Materials and methods

### Ethics statement

The animal experiments conducted in the Institute of Microbiology, Chinese Academy of Sciences (IMCAS) were in strict accordance with the recommendations described in the Guide for the Care and Use of Laboratory Animals of IMCAS Ethics Committee (approval number: APIMCAS2022124). The SARS-CoV-2 challenge experiments on mice and macaques were conducted under the approval from the Animal Ethics Committee of the Wuhan Institute of Virology (WIV), Chinese Academy of Sciences (CAS) (approval number: WIVA42202002-01) according to the National Guidelines on Animal Work in China.

### Plasmids and cells

DNA sequence of SARS-CoV-2 RBDs (Delta: EPI_ISL_2378732, BA.2: EPI_ISL_6640916) were codon-optimized by Stemirna, synthesized by GenScript and subcloned into the vector for *in vitro* mRNA transcription. HEK293T cells (ATCC CRL-3216) and Vero cells (ATCC CCL81) were cultured in Dulbecco’s modified Eagle’s medium (DMEM) supplemented with 10% fetal bovine serum (FBS) at 37°C.

### Animals

C57BL/6 mice (female, 6–8 weeks) were purchased from Beijing Vital River Animal Technology Co., Ltd (licensed by Charles River) and were housed in a specific-pathogen-free (SPF) mouse facilities in IMCAS with temperature-, humidity- and light cycle-control (20 ± 2°C; 50 ± 10%; light, 7:00–19:00; dark, 19:00–7:00). Rhesus macaques (9 females and 9 males, 5~8 years-old) were purchased from Hubei Topgene Biotechnology Co., Ltd. All macaques are in good health and are not involved in other experimental procedure. These macaques were housed in the laboratory animal facilities in Wuhan Institute of Virology (WIV), Chinese Academy of Sciences (CAS) for immunization and SRAS-CoV-2 challenge. All macaques were allowed free access to water and standard diet and provided with a 12-hour light and dark cycle (temperature: 21.0–24.3°C, humidity: 46.5%-100%). SARS-CoV-2 challenge and neutralizing antibody titration assays were performed with approval under Biosafety Level 3 (BSL3) and ABSL3 conditions by the Institutional Biosafety Committee of WIV.

### Preparation of mRNA

Messenger RNA of SWIM516 vaccine was transcribed *in vitro* using linearized plasmid encoding Delta-BA.2 RBD dimer as template. The Delta-BA.2 RBD dimer was flanked by the 5’ and 3’ untranslated regions and a 120 nt poly-A tail. *In vitro* transcription (IVT) and 5’-capping were conducted in one step using one-step mRNA IVT kit (Hongene Biotech, China). Fifty percent of UTP was substituted with 1-methylpseudourine-5’-triphosphate during transcription. Then, mRNA was treated with Antarctic Phosphatase to remove residual 5’-triphosphates (37°C, 30 min) and purified using column chromatography. Capped mRNA was dissolved in 25 mM sodium acetate (pH 5.2) and stored at -80°C until use.

### Encapsulation of mRNA by LPP or LNP

For LPP encapsulation, a core complex was first prepared by mixing protamine dissolved in 25 mM sodium acetate (pH 5.2) with mRNA diluted in 10 mM citrate buffer (pH 4.0) in a volume ratio of 1:5 (protamine: mRNA), and then incubated for 30 min at room temperature. Next, ionizable lipid, DOPE (Avanti Polar Lipids, USA), cholesterol (A.V.T Pharmaceutical, China) and mPEG-DMG (Avanti Polar Lipids, USA) were dissolved in ethanol using previously optimized molecular ratio [39], and then combined with the core complexes at a ratio of 3:1 using Inano E platform (Micro & Nano Technology Inc, China). For LNP encapsulation, ionizable cationic lipid SM102, DSPC, cholesterol and DMG-PEG2000 purchased from A.V.T (A.V.T Pharmaceutical, China) were mixed at a ratio of 50:10:38.5:1.5 (mol/mol), and then combined with mRNAs diluted in sodium acetate at pH 4.0 using the Inano E platform (Micro & Nano Technology Inc, China). Finally, the LNPs or LPPs were dialyzed against PBS (pH 7.4), concentrated using ultra centrifugal filters (EMD Millipore, USA), filtered through a 0.22-μm filter and stored at 4°C until further use.

### *In vitro* characterization of LPPs

LPPs were diluted into PBS for determining size distribution and zeta potential. Zeta potentials were measured by diluting LPPs into folded capillary zeta cell at pH 4.3 or pH 7.2 and loaded into Zetasizer Pro (Malvern Panalytical, UK). Particle size distribution was measured with dynamic light scattering. For cryo-electron microscopy, LPPs were transferred onto a glow-discharged ultrathin carbon-coated copper grid, blotted for 2 s with filter paper in FEI Vitrobot Mark IV (Thermo Fisher Scientific, USA), followed by quick plugging into liquid ethane. Frozen grids were loaded into a Talos transmission electron microscope (Thermo Fisher Scientific, USA) equipped with a field emission gun operated at 200 kV. Images were recorded on a direct electron detector (ED20). For *in vivo* imaging, mRNAs encoding luciferase were encapsulated by LNP or LPP and injected in both legs of BALB/c mice (10 μg/mouse). Bioluminescence image of mice was captured using IVIS Spectrum Imaging System (PerkinElmer, USA) at 6, 12, 24 and 48 h post-injection.

### mRNA transfection and Western blot

HEK293T cells were transfected with TransIT-mRNA (Mirus Bio, USA). Basically, for naked mRNA, 1 μg was added to 100 μl serum-free Opti-MEM together with TransIT-mRNA reagent (2 μl) and booster reagent (2 μl). The complex was incubated for 3 min before added dropwise to 5 × 10^5^ cells cultured in complete medium in 12-well plate. For LPP encapsulated mRNA vaccine, 1 μg was added directly in complete medium in 12-well plate. Supernatants were collected 36 h post transfection and stored at -20°C until use. For western blot, supernatant samples were combined with loading buffer with dithiothreitol, separated by 10% SDS-PAGE and transferred to Polyvinylidene difluoride (PVDF) membrane using a semi-dry apparatus (WIX Technology, China). Then, the membrane was blocked with 5% non-fat milk diluted in TBS-T buffer, blotted with SARS-CoV-2 Spike/RBD primary antibody (Sino Biological, China) for 1h and goat anti-rabbit IgG-HRP secondary antibody (Easybio, China) for 1 h. Finally, the membrane was developed using Beyotime BeyoECL Plus (Beyotime Biotech, China).

### Evaluation of the immunogenicity

All vaccines were immunized by injecting female mice aged 6–8 weeks via the i.m. route. For evaluating the immunogenicity in naïve mice, groups of female C57BL/6 mice (n = 10) were immunized with two doses of mRNA vaccine (10 μg/mouse per dose) or PBS as sham on day 0 and day 14. Blood samples were collected on day 28, splenocytes were prepared on day 28 immediately after sacrifice. For evaluating the immunogenicity of booster shots, groups of female C57BL/6 mice (n = 8) were immunized with three shots (day 0, day 21 and day 35) or four shots (day 0, day 21, day 35 and day 49) of inactivated, protein subunit or mRNA vaccines. The low/high dose per mouse for inactivated, protein subunit and mRNA vaccines are 0.65 U/2.6 U, 2.5 μg/10 μg and 2.5 μg/10 μg, respectively. Blood samples were collected on day 49 (three dose) and day 63 (four dose), splenocytes were prepared at the same day as blood samples immediately after sacrifice. For the side-by-side comparison of LNP and LPP, groups of female BALB/c mice (n = 5) were immunized with two shots (5 μg/mouse per dose) of PT-PT RBD dimer mRNA vaccine with a 14-day interval. Spleens and blood samples were collected on day 21 and day 28, respectively. All blood samples were further centrifuged and the serum in supernatants were stored at -80°C until use. All splenocytes were homogenized with a tissue grinder in 1 ml of serum-free DMEM, filtered with a 40 μm cell strainer (Corning, USA), followed by lysis of red blood cell with red blood cell lysis buffer (Solarbio Life Science, China). Splenocytes were counted using Celldrop FL automated cell counter (DeNovix, USA) after staining with 0.4% trypan blue solution. Live splenocytes were then immediately used for Intracellular cytokine staining (ICS) assay and Enzyme-linked immunospot (ELISpot) assay.

### Evaluation of protective efficacy in mice

For transient model, C57BL/6 mice (female, 6–8 weeks, n = 5) were immunized with two doses of mRNA vaccine (10 μg/mouse per dose) or PBS as sham on day 0 and day 14, followed by infection of recombinant type 5 adenovirus expressing hACE2 via i.n. route at 42. Viral challenge with SARS-CoV-2 Delta or BA.1 virus (1×10^5^ TCID_5_) were conducted five days later on day 47. On day 54, lung, trachea and turbinal tissues of each mouse were harvested for examination of viral load. For stable model, human ACE2 transgenic mice (female, 6–8 weeks, n = 5) were immunized with two doses of mRNA vaccine (3 μg or 10 μg/mouse per dose) or PBS as sham on day 0 and day 14, followed by viral challenge with SARS-CoV-2 Delta or BA.1 virus (1×10^5^ TCID_5_) on day 33. Lung and trachea were collected on day 38 for examination of viral load and pathological damages.

### Evaluation of protective efficacy in rhesus macaques

Rhesus macaques (n = 6) were immunized with clinical-grade SWIM516 mRNA vaccine (Low dose: 25 μg/animal per dose as; High dose: 45 μg/animal per dose) or Placebo on day 0 and day 21, followed by collection of blood samples on day 28, day 35 and day 42. Viral challenge with SARS-CoV-2 BA.1 virus (1×10^5^ TCID_5_) were conducted on day 42 via endotracheal intubation. Seven days post infection (day 49), animals were euthanized and dissected for pathological examination of lungs. Tissue samples were collected from the left lung (upper lobe, middle lobe and inferior lobe) and the right lung (upper lobe, middle lobe, inferior lobe and accessory lobe) for viral load and histopathology.

### Enzyme-linked immunosorbent assay (ELISA)

The recombinant proteins of SARS-CoV-2 PT, Delta, BA.1, BA.2, BA.2.12.1, BA.2.75, BA.3, BA. 5 or BF.7 RBDs were purchased from GenScript. ELISA plates (Corning, USA) were coated overnight with each RBD (2 μg/ml) in 0.05 M carbonate-bicarbonate buffer, pH 9.6. Then, the plates were blocked in 5% non-fat milk diluted in PBS-T buffer. Serum samples were subjected to a three-fold serial dilution starting from 1:20. After adding diluted serum to each well, the plates were incubated for 1 h at 37°C. Goat anti-mouse IgG-HRP antibody (Santa Cruz, USA) was added to plates as secondary antibody and incubated for 1 h at 37°C. The plates were developed with 3,3′,5,5′-tetramethylbenzidine (TMB) substrate (Promega, USA). After stopping reaction with 2M hydrochloric acid, the absorbances at 450 nm and 630 nm were measured using a microplate reader (Agilent Technologies, USA). Absorbance values were calculated by subtracting the absorbance at 630 nm from that at 450 nm of the same well. Endpoint titers were defined as the highest reciprocal dilution of serum to yield an absorbance greater than 2.1-fold of the background values. Antibody titer below the limit of detection was determined as one third of the detection limit.

### Pseudovirus neutralization assay

Vesicular Stomatitis Virus (VSV)-backbone SARS-CoV-2 pseudotyped viruses of examined variants were purchase from Vazyme Biotech (SARS-CoV-2-Fluc WT, DD1502; SARS-CoV-2-Fluc Delta, DD1554; SARS-CoV-2-Fluc BA.1, DD1568; SARS-CoV-2 BA.2, DD1569; SARS-CoV-2 BA.2.12.1, DD1577; SARS-CoV-2 BA.2.75, DD1581; SARS-CoV-2 BA.3, DD1574; SARS-CoV-2 BA.5, DD1576; SARS-CoV-2 BF.7, DD1589). For neutralization assay, the heat-inactivated (56°C, 30 min) serum samples were subjected to a three-fold serial dilution started from 1:60. Each pseudotyped virus (650 TCID_50_/well) was mixed with equal volume of serially diluted sera and incubated at 37°C for 1 h, and then 100 μl mixture was added onto pre-plated Vero cells in 96-well plate. After 15 h incubation, the transducing units (TU) numbers were calculated on a CQ1 confocal image cytometer (Yokogawa, Japan). Neutralization titers were calculated as EC50 titers by Reed-Muench method. Values below the limit of detection were determined as one third of the starting dilution factor.

### Intracellular cytokine staining (ICS) assay

For ICS assay, fresh mouse splenocytes were added into 96-well plate (1 × 10^6^ cells/well). Cells were stimulated with each peptide pool (2 μg/ml for each peptide) for 3 h, and incubated with Golgiplug (BD Biosciences, USA) for 6 h at 37°C. Then, cells were harvested, blocked with recombinant CD16/32 protein, and stained with PE/Cy7-conjugagted anti-CD3, APC/Cy7 conjugated anti-CD4 and PerCP/Cy5.5 conjugated anti-CD8α antibodies (Biolegend, USA). After fixation and permeabilization by permeabilizing buffer (BD Biosciences, USA), cells were further stained with PE conjugated anti-IFN-γ antibody (Biolegend, USA). Cells were analyzed by flow cytometric analysis through a BD LSRFortessa flow cytometer (BD Biosciences, USA) with high-throughput system. Data were analyzed using FlowJo 10.0 (BD Biosciences, USA).

### Enzyme-linked immunospot (ELISpot) assay

For ELISpot assay, flat-bottom 96-well plates were pre-coated with 10 g/ml anti-mouse IFNγ Ab (BD Biosciences, USA) overnight at 4°C and blocked for 2 h at RT. Fresh mouse splenocytes were added into the pre-coated 96-well plates (4 × 10^5^ cells/well) and stimulated with each peptide pool (2 μg/ml for each peptide) for 20 h. Negative control wells were not stimulated with peptide pool. Phytohemagglutinin (PMA) was added to positive control wells. After stimulation, cells were removed from plates and the plates were probed with biotinylated IFNγ antibody, streptavidin-HRP conjugated antibody and 3-amino-9-ethylcarbazole (AEC) substrate. The development was stopped by thoroughly rinsing samples with deionized water when spots became visually observable. Finally, the numbers of spots were determined using CTL ImmunoSpot S6 Analyzer (CTL, USA) and image analysis software Immuno Capture 6.5.0 (Cellular Technology, USA).

### Plaque reduction neutralization test (PRNT) assay

Serum sample from each rhesus macaque was heat inactivated (56°C, 30 min) and subjected to a three-fold serial dilution started at 1:50 using DMEM. Then, diluted sera were mixed with equal volume of SARS-CoV-2 BA.1 (1000 PFU/mL) and incubate for 1 h at 37°C. Then, the serum-virus mixtures were added on pre-plated Vero cells (2×10^5^ cells/well) and incubated for 1 h at 37°C. After incubation, the inoculum was removed. The cells were washed once by PBS, added 1ml DMEM mixed with 0.8% Carboxymethylcellulose and cultured for 96 h. After washing by PBS, plates were fixed with 8% Paraformaldehyde (Solarbio Life science) for 1 h and stained with 0.05% crystal violet overnight. Plaques were captured and calculated by CTL ImmunoSpot S6 Analyzer (CTL, USA) and image analysis software Immuno Capture 6.5.0 (Cellular Technology, USA). The half maximal inhibitory concentration (IC50) was calculated based on plaque numbers by GraphPad Prism 9.0 (GraphPad Software Inc., USA).

### Quantitation of viral load in rhesus macaques

Rhesus macaque lungs were collected as described above and fixed in neutral formalin. Lung samples were weighed, added into serum-free DMEM (1,10 W/V) and grinded with tissue homogenizer. Supernatants were isolated by centrifugation (4500 rpm, 30 min). Viral RNA was extracted with QIAamp Viral RNA Mini Kit (Qiagen, USA) as template of RT-PCR. Detection of SARS-CoV-2 was conducted with HiScript II One Step qRT-PCR SYBR Green Kit (Vazyme, China) on the ABI StepOne Real-Time PCR system (Thermo Fisher Scientific, USA) with the following conditions: 50°C for 3 min and 95°C for 30 s, 40 cycles of amplification at 95°C for 10 s and 60°C for 30 s. The following primers were used: forward 5’-CAATGGTTAAGGCAGG-3’; reverse 5’-CTCAAGGTCTGGATCACG-3’. Viral load (copies/mL) in each sample was calculated based on standard curve.

### Histopathology analysis of lungs

Mice and rhesus macaque lung samples were collected with the method described above. Samples were fixed in neutral formalin and then stained with hematoxylin and eosin for histopathology analysis. Histopathological changes of all collected tissues were graded by a double-blind evaluation, including inflammation and structural changes. Scores 0, 1, 2 and 3 indicate no, mild, medium and severe histopathological damage, respectively.

### Statistical analysis

For ELISA, pseudotyped virus neutralization and live virus PRNT assay, data are presented as geometric mean ± 95% confidence interval (CI). For ICS assay, ELISpot and quantitative PCR, data are presented as mean ± standard deviation (SD). Statistical significances were calculated by Mann-Whitney test or two-tailed unpaired T test (*, p<0.05; **, p<0.01; ***, p<0.001; ****, p<0.0001). All graphs and statistical analyses were generated with GraphPad Prism 9.0 (GraphPad Software Inc., USA).

## Supporting information

S1 Table Evaluation of the histopathological damage in the lungs of hACE2 transgenic mice. The Delta and BA.1 in sample names indicate mice challenged with Delta and BA.1 live virus, respectively. The sham, low and high indicate vaccinated dosage of 0, 3 μg and 10 μg, respectively. Scores 0, 1, 2 and 3 indicate no, mild, medium and severe histopathological damage, respectively.
(XLSX)

S2 Table Evaluation of the histopathological damage in the lungs of unvaccinated rhesus macaques. Scores 0, 1, 2 and 3 indicate no, mild, medium and severe histopathological damage, respectively. (UL: upper left; ML: middle left; LL: lower left; UR: upper right; MR: middle right; LR: lower right).
(XLSX)

S3 Table Evaluation of the histopathological damages in the lungs of rhesus macaques vaccinated with low dose SWIM516. Scores 0, 1, 2 and 3 indicate no, mild, medium and severe histopathological damage, respectively. (UL: upper left; ML: middle left; LL: lower left; UR: upper right; MR: middle right; LR: lower right).
(XLSX)

S4 Table Evaluation of the histopathological damage in the lungs of rhesus macaques vaccinated with high dose SWIM516. Scores 0, 1, 2 and 3 indicate no, mild, medium and severe histopathological damage, respectively. (UL: upper left; ML: middle left; LL: lower left; UR: upper right; MR: middle right; LR: lower right).
(XLSX)

S1 Fig Comparison of the immunogenicity between LNP- and LPP-encapsulated mRNA vaccines. **(a)** Schematic of the SARS-CoV-2 prototype RBD-dimer mRNA vaccine (PP). (**b**) Mice immunization and sample collection schedule. Groups of BALB/c mice immunized with two doses of PP vaccine (5 μg/dose). Serum samples and splenocytes were collected from two different groups of mice on day 28 and day 21, respectively. (**c**) NT_50_ of neutralizing antibodies against the pseudotyped viruses (pVirus) of SARS-CoV-2 prototype. Dashed line indicates starting dilution (40 folds). Data are shown as GMT ± 95% CI. Statistical significances were calculated by the Mann-Whitney test. (**d**) ELISpot assay quantifying the IFNγ-secreting splenocytes after re-stimulation by RBD peptide pool of SARS-CoV-2 prototype. Data are shown as means ± SD. Statistical significances were calculated by unpaired T test.
(TIF)

S2 Fig *In silico* prediction of MHC class I binding peptides in the RBDs of SARS-CoV-2 variants. Peptides (9-mer) with high binding affinity to MHC class I in C57BL/6 mice (H-2-Kb and H-2-Db alleles) were predicted using NetMHC 4.0 (https://services.healthtech.dtu.dk/services/NetMHC-4.0/). Dashed boxes indicate conserved peptides with high affinity to MHC class I. Numbers below dashed boxes represent the predicted binding affinity of the indicated peptide.
(TIF)
