# Supplementary figures and images for: Broad-spectrum Delta-BA.2 tandem-fused heterodimer mRNA vaccine delivered by lipopolyplex
Source: PLoS Pathog. 2024 Apr 1;20(4):e1012116. doi: 10.1371/journal.ppat.1012116 (PMC11008869; doi:10.1371/journal.ppat.1012116)

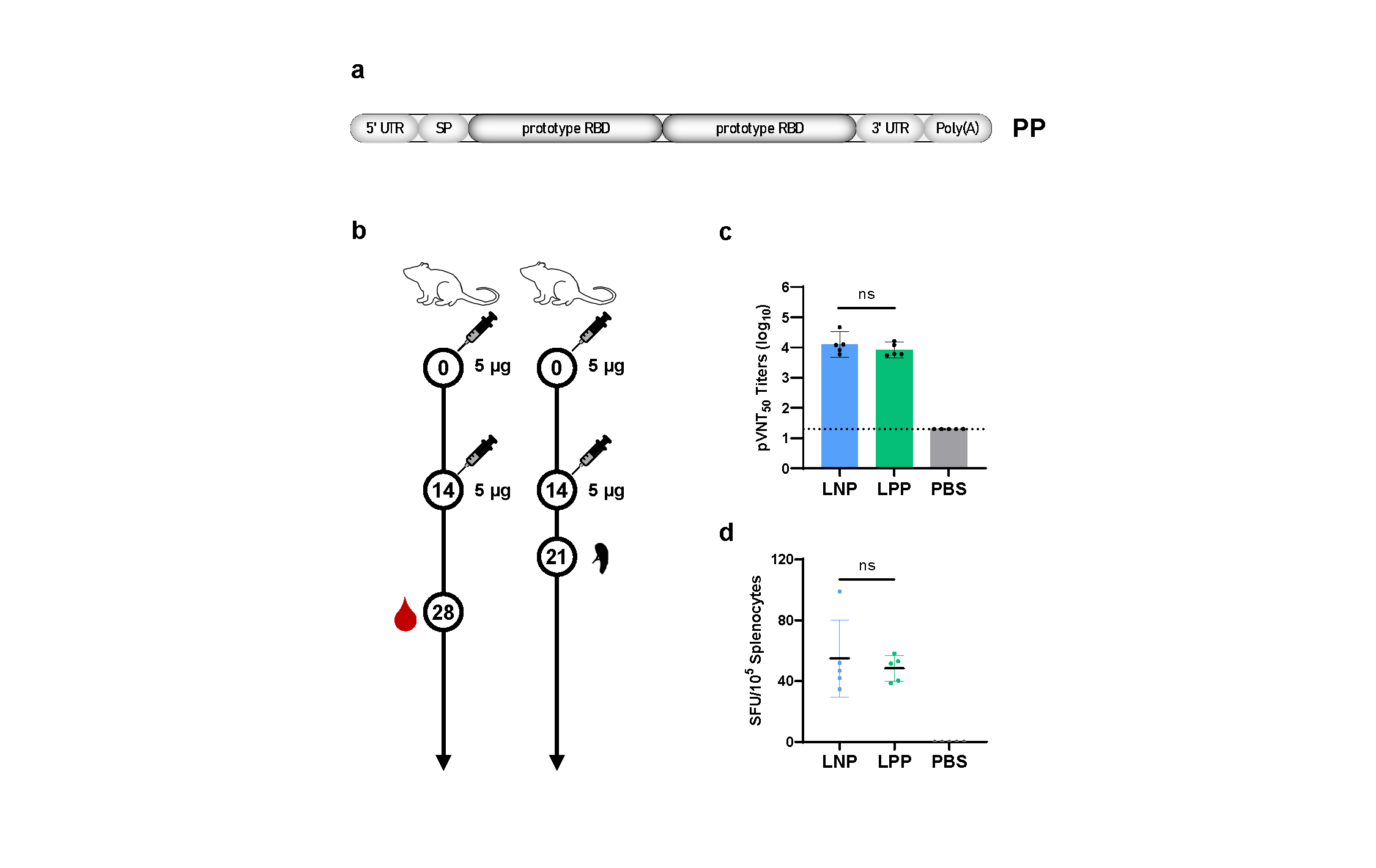

Supplement: S1 Fig — (a) Schematic of the SARS-CoV-2 prototype RBD-dimer mRNA vaccine (PP). (b) Mice immunization and sample collection schedule. Groups of BALB/c mice immunized with two doses of PP vaccine (5 μg/dose). Serum samples and splenocytes were collected from two different groups of mice on day 28 and day 21, respectively. (c) NT50 of neutralizing antibodies against the pseudotyped viruses (pVirus) of SARS-CoV-2 prototype. Dashed line indicates starting dilution (40 folds). Data are shown as GMT ± 95% CI. Statistical significances were calculated by the Mann-Whitney test. (d) ELISpot assay quantifying the IFNγ-secreting splenocytes after re-stimulation by RBD peptide pool of SARS-CoV-2 prototype. Data are shown as means ± SD. Statistical significances were calculated by unpaired T test. (TIF) [file ppat.1012116.s005.tif]

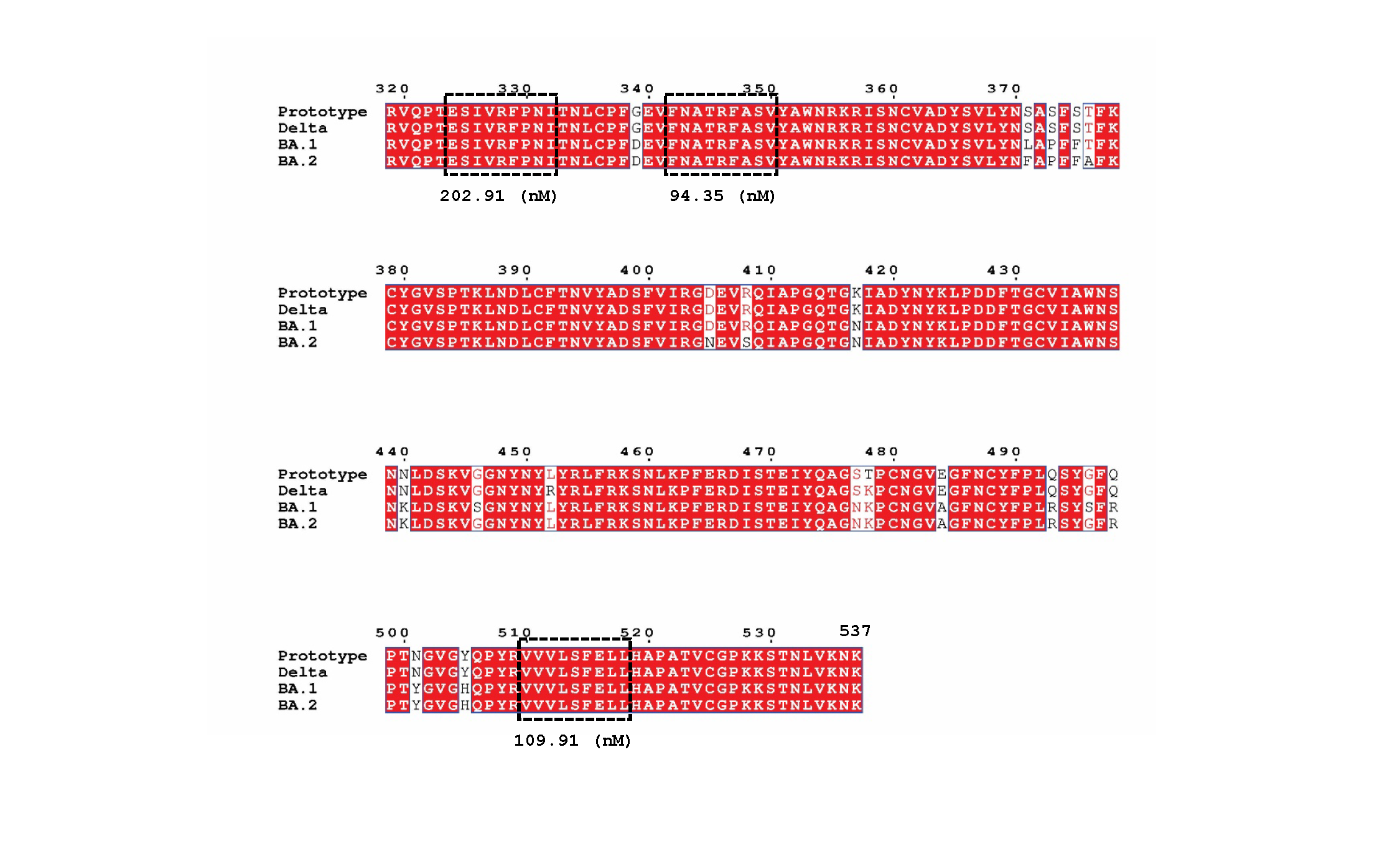

Supplement: S2 Fig — Peptides (9-mer) with high binding affinity to MHC class I in C57BL/6 mice (H-2-Kb and H-2-Db alleles) were predicted using NetMHC 4.0 (https://services.healthtech.dtu.dk/services/NetMHC-4.0/). Dashed boxes indicate conserved peptides with high affinity to MHC class I. Numbers below dashed boxes represent the predicted binding affinity of the indicated peptide. (TIF) [file ppat.1012116.s006.tif]
